# Fast and Accurate Plane Wave and Color Doppler Imaging with the FOCUS Software Package

**DOI:** 10.3390/s25144276

**Published:** 2025-07-09

**Authors:** Jacob S. Honer, Robert J. McGough

**Affiliations:** Department of Electrical and Computer Engineering, Michigan State University, East Lansing, MI 48824, USA; honerja1@msu.edu

**Keywords:** plane wave imaging, color Doppler imaging, B-mode imaging, synthetic aperture imaging, biomedical ultrasound, fast nearfield method (FNM), fast object-oriented C++ ultrasound simulator (FOCUS), spatial impulse response

## Abstract

A comprehensive framework for ultrasound imaging simulations is presented. Solutions to an inhomogeneous wave equation are provided, yielding a linear model for characterizing ultrasound propagation and scattering in soft tissue. This simulation approach, which is based upon the fast nearfield method, is implemented in the Fast Object-oriented C++ Ultrasound Simulator (FOCUS) and is extended to a range of imaging modalities, including synthetic aperture, B-mode, plane wave, and color Doppler imaging. The generation of radiofrequency (RF) data and the receive beamforming techniques employed for each imaging modality, along with background on color Doppler imaging, are described. Simulation results demonstrate rapid convergence and lower error rates compared to conventional spatial impulse response methods and Field II, resulting in substantial reductions in computation time. Notably, the framework effectively simulates hundreds of thousands of scatterers without the need for a full three-dimensional (3D) grid, and the inherent randomness in the scatterer distributions produces realistic speckle patterns. A plane wave imaging example, for instance, achieves high fidelity using 100,000 scatterers with five steering angles, and the simulation is completed on a personal computer in a few minutes. Furthermore, by modeling scatterers as moving particles, the simulation framework captures dynamic flow conditions in vascular phantoms for color Doppler imaging. These advances establish FOCUS as a robust, versatile tool for the rapid prototyping, validation, and optimization of both established and novel ultrasound imaging techniques.

## 1. Introduction

For simulations of ultrasound imaging that model linear transducer arrays and phased arrays, several analytically equivalent methods are applied to computations of transient pressures in the nearfield and far-field regions. One approach for computing transient pressures evaluates the spatial impulse response numerically, where the spatial impulse response describes the transient pressure for a specified transducer geometry at all points in space for an impulsive input [1,2]. The spatial impulse response is implemented in the time domain in Field II [3] and in the frequency domain in the MUST toolbox [4]. One advantage of the spatial impulse response is that analytical expressions for the spatial impulse response are available for several different transducer geometries, where these analytical expressions are valuable for developing a more intuitive understanding of transient diffraction [1,2,5,6,7,8,9]. Another important advantage is that the spatial impulse response accelerates imaging simulations applied to hundreds of thousands of scatterers by eliminating the need for a three-dimensional (3D) rectangular grid. Other methods for computing transient pressures utilize 3D finite difference methods or pseudo-spectral methods. These are the methods implemented in FullWave [10] and k-Wave [11], respectively. Although finite difference and pseudo-spectral methods provide opportunities to model other acoustic phenomena, 3D ultrasound simulations with these numerical methods are much more time-consuming than those based on the spatial impulse response [10,12].

The fast nearfield method is another numerical approach that retains the computational advantage of defining unstructured scatterer distributions to enable rapid 3D simulations of ultrasound imaging. The fast nearfield method is implemented in FOCUS, the “Fast Object-oriented C++ Ultrasound Simulator” (https://www.egr.msu.edu/~fultras-web/ (accessed on 15 May 2025)), for both continuous-wave [13,14] and transient [15] pressure calculations. FOCUS supports a variety of transducer shapes, including rectangular, circular, spherical, flat ring, and spherical ring transducers [16]. FOCUS provides the software infrastructure for one-way calculations and B-mode imaging simulations [17]. FOCUS is free software; example scripts and documentation are available on the FOCUS website. Transient pressure calculations in FOCUS are accelerated by separating the temporal and spatial components of rapidly converging single integral expressions. This transformation converts the transient pressure calculations into the superposition of a small number of rapidly converging spatial integrals, each weighted by time-dependent factors. This approach, known as time–space decomposition [15] for predefined excitation functions and frequency-domain time–space decomposition [18] for arbitrary excitations, substantially reduces the computation time. FOCUS also supports the angular spectrum approach [19], a frequency-domain continuous-wave simulation algorithm that propagates a source plane through the Fourier domain to a destination plane, yielding a method that is particularly effective for large, continuous-wave volumetric (3D) pressure field computations. In addition, FOCUS extends simulation capabilities beyond linear propagation by supporting nonlinear phenomena through both the continuous-wave and transient Khokhlov–Zabolotskaya–Kuznetsov (KZK) equations for circular and spherical transducers [16].

Although computation time and accuracy are important considerations for simulations of ultrasound imaging, additional supporting features are also necessary. For instance, support for various transducer shapes and array configurations is essential. In addition, the software should ideally simulate different imaging scenarios with a minimal amount of additional programming. Whereas basic functionalities, such as simulations of B-mode imaging, are included in many of the aforementioned software packages, direct support for advanced simulations such as plane wave imaging [20] and color Doppler (color flow) imaging [21] is lacking in most of these programs. For example, Field II provides a color Doppler example simulating a carotid artery using B-mode imaging, but Field II lacks support for plane wave imaging. MUST offers support for plane wave and color Doppler imaging, but the provided color Doppler imaging examples rely on experimental RF data rather than simulated RF data. The FullWave and k-Wave software packages do not presently provide any examples of plane wave or color Doppler imaging. Although FOCUS provides extensive support for a wide variety of transducer shapes and array geometries, FOCUS also presently lacks support for plane wave and color Doppler imaging.

To address this deficiency while leveraging the computational advantages of the fast nearfield method, a comprehensive framework is presented for plane wave and color Doppler imaging within the FOCUS software package. An introduction to the enhanced computational framework begins with an overview of a time-domain pulse-echo model for ultrasound propagation and scattering in soft tissue, where solutions are obtained by temporally convolving two spatial impulse responses [22]. Building on this foundation, an improved ultrasound imaging model based on the fast nearfield method is described. Methods for synthetic aperture [23], B-mode [24], and plane wave imaging are developed from this improved model, including the generation of RF data and the receive beamforming techniques applied. Additionally, background on color Doppler imaging is provided. Simulation results for each imaging method showcase several examples of this new functionality. The convergence of the improved ultrasound imaging model based on the fast nearfield method, in comparison to the analytically equivalent pulse-echo model with spatial impulse responses and Field II, is also examined.

## 2. Methods

### 2.1. An Enhanced Pulse-Echo Model with the Fast Nearfield Method

The pulse-echo model for ultrasound propagation and scattering in soft tissue is based on time-dependent Green’s function solutions to an inhomogeneous wave equation [25], providing a linear framework for characterizing ultrasound propagation and scattering. Specifically, the model describes the signal generated when an incident time-varying pressure field from a single transducer reflects off an individual scatterer within a medium and is subsequently captured by another transducer [22]. This interaction is mathematically described with spatial impulse responses by

(1) $$p_{r}\left({\vec{r}}_{j},t\right)=\frac{\rho_{0}}{2c^{2}}\frac{\partial v\left(t\right)}{\partial t}*\frac{{\partial h}_{T}\left({\vec{r}}_{i},{\vec{r}}_{n},t\right)}{\partial t}*\frac{{\partial h}_{R}\left({\vec{r}}_{j},{\vec{r}}_{n},t\right)}{\partial t}a_{s}\left({\vec{r}}_{n}\right),$$

where c is the sound speed, $\rho_{0}$ is the density of the medium, $v\left(t\right)$ is the time-varying normal velocity of the transmitting transducer face over time t, and $h_{T}$ and $h_{R}$ are the spatial impulse responses from the transmitting and receiving transducers centered at ${\vec{r}}_{i}$ and ${\vec{r}}_{j}$, respectively. The scatterer location is indicated by ${\vec{r}}_{n}$, the time is t, and $a_{s}$ is the scatterer amplitude, which accounts for the inhomogeneities produced by the density perturbations of each scatterer. The ∗ operator denotes temporal convolution. The signal $p_{r}\left({\vec{r}}_{j},t\right)$ is received by the transducer centered at the location ${\vec{r}}_{j}$, where various collections of these received signals constitute the RF data. The model assumes an isotropic, homogenous, non-dissipative medium with independent scatterers. Scatterers are represented as instantaneous, normalized changes in density with $a_{s}\left({\vec{r}}_{n}\right)=∆\rho ({\vec{r}}_{n})$/$\rho_{0}$.

When $v\left(t\right)$ is expressed as the temporal convolution of two well-behaved oscillatory signals, $v\left(t\right)=w_{1}(t)\;*w_{2}(t)$, Equation (1) becomes [17]

(2) $$p_{r}\left({\vec{r}}_{j},t\right)=\frac{\partial }{\partial t}\left\{\frac{1}{2{\rho_{0}c}^{2}}\left[\rho_{0}\;\frac{\partial w_{1}\left(t\right)}{\partial t}*h_{T}\left({\vec{r}}_{i},{\vec{r}}_{n},t\right)\right]*\left[\rho_{0}\;\frac{\partial w_{2}\left(t\right)}{\partial t}\;*\;h_{R}\left({\vec{r}}_{j},{\vec{r}}_{n},t\right)\right]\;a_{s}\left({\vec{r}}_{n}\right)\right\}.$$

The received signal, as described by Equation (2), is now the temporal convolution of the expressions

(3) $$p_{T}\left({\vec{r}}_{i},{\vec{r}}_{n},t\right)=\left[\rho_{0}\;\frac{\partial w_{1}\left(t\right)}{\partial t}*h_{T}\left({\vec{r}}_{i},{\vec{r}}_{n},t\right)\right],$$

(4) $$p_{R}\left({\vec{r}}_{j},{\vec{r}}_{n},t\right)=\left[\rho_{0}\;\frac{\partial w_{2}\left(t\right)}{\partial t}*h_{R}\left({\vec{r}}_{j},{\vec{r}}_{n},t\right)\right],$$

where $p_{T}\left({\vec{r}}_{i},{\vec{r}}_{n},t\right)$ and $p_{R}\left({\vec{r}}_{j},{\vec{r}}_{n},t\right)$ are intermediate expressions that are determined from the spatial impulse responses of the transmitting and receiving transducers, respectively.

The expressions in Equations (3) and (4) describe solutions for time-varying acoustic pressure fields with normal velocities of $w_{1}(t)$ and $w_{2}(t)$, respectively, which can be calculated with the Rayleigh integral, the spatial impulse response, or the fast nearfield method [14]. Among these methods, the fast nearfield method achieves the fastest convergence and lowest error rates. Therefore, replacing the acoustic pressure field formulations in Equations (3) and (4) with those derived from the fast nearfield method is advantageous for numerical calculations. This combination of terms establishes the methodology for pulse-echo calculations with the fast nearfield method, which is expressed as

(5) $$p_{r}\left({\vec{r}}_{j},t\right)=\frac{\partial }{\partial t}\left\{\frac{1}{2{\rho_{0}c}^{2}}p_{T}\left({\vec{r}}_{i},{\vec{r}}_{n},t\right)\;*p_{R}\left({\vec{r}}_{j},{\vec{r}}_{n},t\right)\;a_{s}\left({\vec{r}}_{n}\right)\right\}.$$

While Equation (5) is analytically equivalent to pulse-echo calculations with spatial impulse responses, aliasing of the spatial impulse responses exacerbates numerical inaccuracies [9,13,17]. The fast nearfield method circumvents these issues, thereby achieving improved convergence, particularly in the nearfield and at the focal peak where aliasing of the spatial impulse response is most prevalent.

### 2.2. Incorporating Time Delays

In the pulse-echo model that evaluates spatial impulse responses, time delays are incorporated directly into the spatial impulse response calculations for the transmitting and receiving transducers. The pulse-echo model with the fast nearfield method similarly incorporates time delays into the intermediate expressions for the transmitting and receiving transducers according to

(6) $$p_{r}\left({\vec{r}}_{j},t\right)=\frac{\partial }{\partial t}\left\{\frac{1}{2{\rho_{0}c}^{2}}p_{T}\left({\vec{r}}_{i},{\vec{r}}_{n},t+\tau_{i}\right)\;*p_{R}\left({\vec{r}}_{j},{\vec{r}}_{n},t+\tau_{j}\right)\;a_{s}\left({\vec{r}}_{n}\right)\right\},$$

where $\tau_{i}$ and $\tau_{j}$ are the time delays of the transmitting and receiving transducers, respectively. In most simulations, the RF data starts at t=0. However, if negative time delays are defined, then the RF data starts at an earlier time.

### 2.3. Ultrasound Imaging Simulations

**Figure 1:**
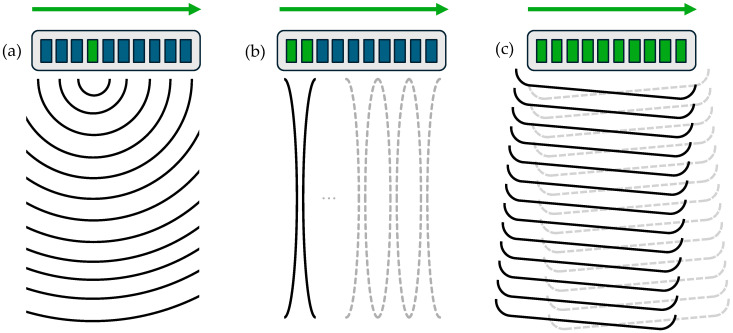
Schematic representations of (**a**) synthetic aperture imaging, (**b**) B-mode imaging, and (**c**) plane wave imaging. Green elements indicate actively transmitting transducer elements, while blue elements represent inactive (non-transmitting) elements. The green arrows denote the direction of scanning or beam steering for subsequent ultrasonic beams or scans.

Simulations of ultrasound imaging with the fast nearfield method are readily extended to synthetic aperture imaging, B-mode imaging, and plane wave imaging. The mathematical structure of each imaging method is outlined in the following sections. Figure 1 provides schematic representations of each imaging method to clarify the distinction between these for the ensuing mathematical formulations.

#### 2.3.1. Synthetic Aperture Imaging

The RF data for synthetic aperture imaging is generated by computing the contribution from a single transmitting transducer and individually convolving that contribution with the contributions from each receiving transducer. This is expressed in terms of the intermediate terms calculated using the fast nearfield method as

(7) $$p_{r}\left({\vec{r}}_{j},t\right)=\frac{\partial }{\partial t}\left\{\frac{1}{2{\rho_{0}c}^{2}}\sum_{n=1}^{N}p_{T}\left({\vec{r}}_{i},{\vec{r}}_{n},t+\tau_{i}\right)*p_{R}\left({\vec{r}}_{j},{\vec{r}}_{n},t+\tau_{j}\right)\;a_{s}\left({\vec{r}}_{n}\right)\right\},\forall i=\left[1\;\ldots \;M\right],\;\forall j=\left[1\;\ldots \;M\right].$$

The notation $\forall i=\left[1\;\ldots \;M\right]$ and $\forall j=\left[1\;\ldots \;M\right]$ indicates that the operation is performed for each transmitting transducer indexed by i within the set of M transducers and for each receiving transducer indexed by j within the same set. Thus, the pulse-echo signals are computed for all pairwise combinations of transmitting and receiving transducers. These pairwise pulse-echo signals are scaled by the respective scatterer amplitudes and summed for all scatterers within a medium.

#### 2.3.2. B-Mode Imaging

To generate a simulated B-mode image, small sub-apertures of a larger transducer array are sequentially focused and excited. Using this approach, the RF data for a single A-line is generated by extending the pulse-echo pressure calculations with the fast nearfield method to

(8) $$p_{r}\left({\vec{r}}_{j},t\right)=\frac{\partial }{\partial t}\left\{\frac{1}{2{\rho_{0}c}^{2}}\sum_{n=1}^{N}\left[\sum_{i=1}^{M}p_{T}\left({\vec{r}}_{i},{\vec{r}}_{n},t+\tau_{i}\right)\right]\;*\left[\sum_{\;j=1}^{M}p_{R}\left({\vec{r}}_{j},{\vec{r}}_{n},t+\tau_{j}\right)\right]\;a_{s}\left({\vec{r}}_{n}\right)\right\}.$$

The outer summation is evaluated over N scatterers, while the inner summations are evaluated over the M transducers within each sub-aperture. By computing and beamforming the intermediate terms before convolution, the computational load for generating a single A-line is reduced.

#### 2.3.3. Plane Wave Imaging

In simulations of plane wave imaging, instead of focusing and beamforming small sub-apertures within a larger array, one or more unfocused plane waves are transmitted across the entire array. To adapt the pulse-echo imaging model with the fast nearfield method to plane wave imaging, the RF data is calculated with

(9) $$p_{r}\left({\vec{r}}_{j},t\right)=\frac{\partial }{\partial t}\left\{\frac{1}{2{\rho_{0}c}^{2}}\sum_{n=1}^{N}\left[\sum_{i=1}^{M}p_{T}\left({\vec{r}}_{i},{\vec{r}}_{n},t+\tau_{i}\right)\right]\;*p_{R}\left({\vec{r}}_{j},{\vec{r}}_{n},t+\tau_{j}\right)\;a_{s}\left({\vec{r}}_{n}\right)\right\},\forall j=\left[1\;\ldots \;M\right].$$

The received pulse-echo signals are calculated element-wise across all receiving transducers. This approach minimizes the number of convolution operations by beamforming the intermediate terms for the transmitting aperture before convolution with the intermediate terms for the receiving transducers.

### 2.4. Accelerated Pressure Calculations

When simulating multiple synthetic aperture images, B-mode images, or plane wave images, a discrete-time shifting technique is employed to reduce the computation time [26]. This is achieved by calculating each intermediate expression for all scatterers for all transducers with no time delays and storing these intermediate results in computer memory. Then, discrete-time shifting with linear interpolation is performed by applying the appropriate time delay to the stored waveforms for each synthetic aperture image, B-mode image, or plane wave image. The fastest imaging calculations in FOCUS utilize this discrete-time shifting technique.

An approach that mitigates the errors caused by discrete-time shifting upsamples the intermediate calculations by four times the temporal sampling frequency and stores these highly sampled waveforms in memory. Time shifting is then applied to these upsampled waveforms, followed by linear interpolation. The resulting waveforms are then downsampled to the original temporal sampling frequency prior to convolution.

### 2.5. Delay-and-Sum Beamforming

To obtain an ultrasound image, delay-and-sum beamforming is applied to the RF data [27]. Focal depths along the *z*- and *x*-axes are defined. A constant f-number determines which pulse-echo signals are included in the beamforming at each focal depth. Time windowing along the *z*-axis ensures consistent spacing between the focal depths. After calculating the time delays and beamforming the RF data at each focal depth, the beamformed A-line segments are combined at all focal depths. The ultrasound image is then generated by demodulating the data using a Hilbert transform and applying log compression. Compounding data from multiple frames enhances image quality, though this reduces frame rates and increases computation time.

### 2.6. Color Doppler Imaging

To simulate color Doppler imaging, flow velocities are estimated using a 1D autocorrelation phase-based estimator [21,28]. After demodulating the beamformed RF data, some additional filtering is applied to enhance the estimation accuracy. First, mean subtraction across pulse transmissions removes static tissue contributions from the RF data while preserving dynamic signal variations from moving scatterers [29]. Next, the RF data is normalized by the peak signal magnitude and thresholded to suppress noise in regions of low signal strength. Since mean subtraction removes static components of the RF data, any residual signal in these regions of low signal strength generally corresponds to predominantly static tissue and is mainly noise that leads to erroneous phase estimates. Thus, thresholding is necessary to mitigate these errors. The mean phase shift across consecutive transmissions is then computed using a 1D autocorrelation phase-based estimator, and the flow velocity, $v_{z}$, is given by [28]

(10) vz=cfPRF4πfocosθtan−1ImR^r→x,tReR^r→x,t,

where c is the sound speed, $f_{PRF}$ is the pulse repetition frequency, $f_{o}$ is the center frequency of the time-varying normal velocity, θ is the angle of flow relative to the tangent of the transducer array, and $\hat{R}\left({\vec{r}}_{x},t\right)$ represents the mean autocorrelation of the demodulated and filtered RF data at position ${\vec{r}}_{x}$ averaged over successive frames. A Hamming moving spatial average filter is then applied to smooth the velocity estimates, reducing outliers and yielding the smooth flow profiles characteristic of color Doppler imaging [30].

## 3. Results

All simulations are performed on the macOS Sequoia operating system on a MacBook Pro with the Apple M1 Pro chip and 16 GB of RAM. The simulations employed FOCUS version 981, compiled for Apple Silicon Macs.

### 3.1. Imaging Examples

For simulations of synthetic aperture, B-mode, and plane wave imaging, a 128-element linear array of rectangular transducers is evaluated. Each rectangular transducer has a width of 0.3 mm, a height of 7.0 mm, and a kerf of 0.05 mm. The linear array is centered at x=0, y=0,z=0, and the array normal is aligned with the *z*-axis. The time-varying normal velocities for the waveforms $w_{1}\left(t\right)$ and $w_{2}\left(t\right)$, which satisfy $w_{1}\left(t\right)=w_{2}\left(t\right)=\sin\left(2\pi f_{0}t\right)$, are single-cycle sinusoids with center frequencies of 3 MHz. All simulations utilize a temporal sampling frequency of 16 MHz, a sound speed of 1540 m/s, and a density of 1000 kg/m^3^. For the fast nearfield method calculations, six abscissas are defined for Gauss–Legendre quadrature.

**Figure 2:**
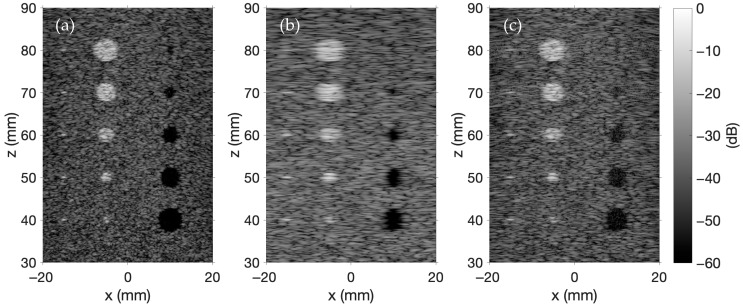
Simulated images of an ultrasound phantom using (**a**) synthetic aperture imaging, (**b**) B-mode imaging, and (**c**) plane wave imaging in FOCUS.

The simulated ultrasound phantom in Figure 2 includes five point targets, five hyperechoic regions, and five anechoic regions. The background is generated by distributing scatterers randomly within a 50 × 10 × 60 mm region 30 mm away from the transducer array. Scatterer amplitudes satisfy a Gaussian distribution with a mean of 0 and a variance of 1. If a randomly distributed scatterer is located in an anechoic region, the scatterer amplitude is set to 0; however, if the scatterer is in a hyperechoic region, the scatterer amplitude is multiplied by 10. The scatterer amplitude of each point target is 20. The simulations in Figure 2 model 100,000 scatterers using a phantom structure that is adapted from a similar script provided by the Field II package [31].

In Figure 2, each image is constructed using 257 A-lines positioned at both the spatial centers of each transducer and the midpoints between transducers. The lateral extent of the ultrasound image ranges from −22.4 mm to 22.4 mm in increments of 0.1764 mm. The axial extent ranges from 30 mm to 90 mm. Figure 2a showcases synthetic aperture imaging, where each transducer is excited individually, and the backscattered pulse-echo signals are captured by all transducers. The delay-and-sum beamforming applies axial focusing along the *z*-axis from 30 mm to 90 mm in 1 mm increments with an f-number of 2, while lateral focusing along the *x*-axis follows the 257 A-line positions spanning from −22.4 mm to 22.4 mm. The final synthetic aperture image is generated by compounding the individual images. Figure 2b is created using B-mode imaging. In this figure, a 64-element sub-aperture array is apodized with a Hanning window and focused to a depth of 60 mm on transmit. Receive focusing is applied at depths of 35, 45, 55, 65, 75, and 85 mm such that each A-line concatenates six segments extending from 30 to 90 mm in 10 mm increments. This process is repeated, shifting the sub-aperture across the array to produce a full set of A-lines. Figure 2c shows an example of plane wave imaging in FOCUS. The plane wave imaging simulation in FOCUS models electronic steering across five evenly spaced transmit angles ranging from −6° to +6°. Delay-and-sum beamforming again applies receive focusing along the *z*-axis from 30 mm to 90 mm in 1 mm increments with an f-number of 2, and along the *x*-axis at 257 focal positions spanning from −22.4 mm to 22.4 mm. Each beamformed transmit–receive RF data pair is then combined to produce the final image. In all three images, each A-line is normalized, demodulated through a Hilbert transform, and log compressed. The normalized, demodulated, and compressed A-lines are then concatenated to produce the final images. The colorbar in Figure 2 shows the display dynamic range in decibels (dB) for each image. That same range is applied uniformly to all of the presented simulated ultrasound images.

**Figure 3:**
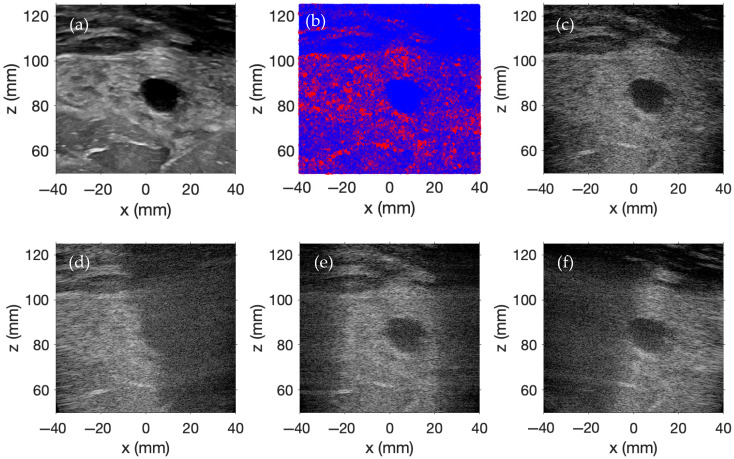
(**a**) Ultrasound image of a female breast with a benign mass, (**b**) randomly distributed scatterers derived from the ultrasound image of a female breast with a benign mass, (**c**) compounded simulated plane wave image of a female breast with a benign mass, and simulated plane wave images corresponding to the steering angles of (**d**) −15°, (**e**) 0°, and (**f**) +15°.

Figure 3 demonstrates plane wave imaging with the same 128-element array that was evaluated in Figure 2. The time-varying normal velocities for the waveforms $w_{1}\left(t\right)$ and $w_{2}\left(t\right)$, which again satisfy $w_{1}\left(t\right)=w_{2}\left(t\right)=\sin\left(2\pi f_{0}t\right)$, are single-cycle sinusoids with center frequencies of 3 MHz. The temporal sampling frequency is 16 MHz, the sound speed is 1540 m/s, and the density is 1000 kg/m^3^.

Figure 3a contains a two-dimensional (2D) ultrasound image of a female breast with a benign mass obtained from the Breast Ultrasound Images Dataset [32], which serves as the scatterer amplitude template for this simulation. One million scatterers are randomly distributed within a 100 × 15 × 100 mm region. Scatterer amplitudes are determined by mapping the *x*–*z* coordinates of each scatterer to the nearest pixel in the 2D grayscale ultrasound image, with a logical mask excluding any points outside the image bounds. Each grayscale pixel value is divided by 100, yielding scaled amplitudes ranging from 0 to 2.55. An exponential transformation of these scaled amplitudes produces strictly positive weights that accentuate bright regions relative to darker areas. Subtraction of the global minimum weight from all weights establishes a zero baseline, and the spectrum of weights is uniformly rescaled to range from 0 to $10^{6}$. Finally, independent Gaussian random samples with a mean of 0 and a variance of 1 are multiplied by the rescaled weights, yielding zero-mean amplitudes with local variance that reflects the grayscale variations of the original ultrasound image. The finalized collection of scatterers, intended to mimic a female breast with a benign mass, is pictured in Figure 3b.

Plane wave imaging is performed across nine evenly spaced transmit angles, ranging from −20° to +20°. Each image is beamformed using delay-and-sum beamforming with receive focusing ranging from 50 mm to 125 mm in the *z*-direction in 1 mm intervals and from −40 mm to 40 mm in the *x*-direction in 0.1566 mm intervals. The f-number for the delay-and-sum beamforming is 1.5. The individual images for the steering angles of −15°, 0°, and +15° are displayed in Figure 3d, Figure 3e, and Figure 3f, respectively. The final compounded plane wave image is shown in Figure 3c. The display dynamic range for each simulated image spans from 0 to −60 dB.

As indicated by the images in the bottom row of Figure 3, each steered plane wave insonifies different areas within the region of interest, and backscattered signals are strongest in regions where the spatial pressure magnitude of the transmitted wavefront is highest. However, by transmitting plane waves at multiple steering angles and coherently compounding the beamformed RF data from each transmission, the entire region is effectively imaged in these simulations. Furthermore, this compounding enhances lateral resolution and contrast by reducing direction-dependent artifacts and by increasing spatial coherence through the reinforcement of consistent echo patterns across steering angles.

**Figure 4:**
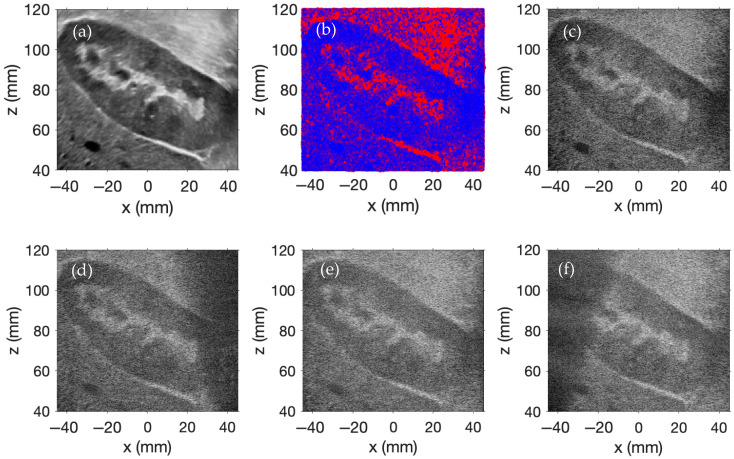
(**a**) Ultrasound image of a human kidney, (**b**) randomly distributed scatterers derived from the ultrasound image of a human kidney, (**c**) compounded simulated plane wave image of a human kidney, and simulated plane wave images corresponding to the steering angles of (**d**) −15°, (**e**) 0°, and (**f**) +15°.

Figure 4 presents a second example of plane wave imaging simulated using FOCUS. A 256-element linear array of rectangular transducers is employed, with each element having a width of 0.3 mm, a height of 7.0 mm, and a kerf of 0.05 mm. The array is centered at x=0, y=0,z=0, with the element normals oriented along the *z*-axis. The waveforms $w_{1}\left(t\right)$ and $w_{2}\left(t\right)$ are defined by $w_{1}\left(t\right)=w_{2}\left(t\right)=\sin\left(2\pi f_{0}t\right)$, where each is a single-cycle sinusoidal waveform with a center frequency of 3 MHz. The temporal sampling frequency is 16 MHz in the simulation. The sound speed is 1540 m/s, and the density is 1000 kg/m^3^.

A 2D ultrasound image of a human kidney from the Kidney Ultrasound Images “Stone” and “No Stone” dataset [33], shown in Figure 4a, serves as the scatterer amplitude scaling template for this simulation. One million scatterers are randomly distributed within a 100 × 15 × 100 mm region. The scatterer amplitudes are assigned using the same method as described in the previous example.

Plane wave imaging is simulated using nine transmit angles evenly distributed from −20° to +20°. Delay-and-sum beamforming is applied to each acquisition with receive focusing along the z-direction from 40 mm to 120 mm with 1 mm increments and along the x-direction from −45 mm to 45 mm with 0.1761 mm increments. Delay-and-sum beamforming is performed with an f-number of 1.5. The beamformed images corresponding to steering angles of −15°, 0°, and +15° are shown in Figure 4d, Figure 4e, and Figure 4f, respectively. The final compounded image, formed by coherently compounding all steered acquisitions, is displayed in Figure 4c. Each simulated image employs a display dynamic range of 0 to −60 dB.

This example again illustrates how coherent compounding improves resolution and contrast in plane wave imaging simulations. Compared to the previous configuration, the longer lateral span of the 256-element array enables broader plane wave coverage, reducing the presence of shadowed or under-illuminated regions in the individual images. As a result, each single-angle acquisition captures more of the region of interest with fewer dark areas. This improved per-angle coverage yields a final compounded image with more uniform brightness, particularly in the lower corners where coverage was previously more limited. The increased strength and consistency of the RF data in these regions contribute to improved resolution and contrast, as spatial features are more effectively reinforced across multiple steered angles—unlike in the previous simulation, where limited coverage resulted in weaker representations of those areas. However, this improvement incurs a computational cost: doubling the number of transducer elements relative to the prior simulation nearly quadruples the simulation time, reflecting the increased memory usage and computational demands associated with a larger transducer array.

**Figure 5:**
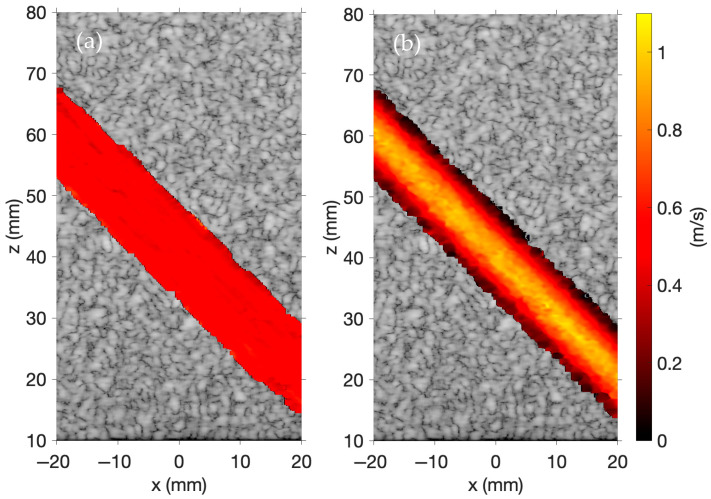
Simulated color Doppler images with (**a**) laminar flow and (**b**) parabolic flow.

To produce the color Doppler images in Figure 5, the time-varying waveforms are given by $w_{1}\left(t\right)=\sin\left(4*2\pi f_{0}t\right)$ and $w_{2}\left(t\right)=\sin\left(2\pi f_{0}t\right)$. These correspond to a four-cycle sinusoidal waveform and a one-cycle sinusoidal waveform, each with a center frequency of 2.5 MHz. Both simulations define a temporal sampling frequency of 20 MHz, a sound speed of 1540 m/s, and a density of 1000 kg/m^3^. The 256-element linear array of rectangular transducers defined for these simulations is centered at x=0, z=0,y=0. The transducer width is 0.3 mm, the height is 7 mm, and the kerf is 0.05 mm.

A set of 50,000 moving scatterers is generated to mimic the flow dynamics of a carotid artery. These scatterers are randomly distributed within a 3D vessel with a radius of 5 mm, oriented at a 45° angle, and positioned 40 mm away from the transducer array. Additionally, 50,000 static scatterers are also generated, randomly distributed within a 60 × 10 × 70 mm region, excluding the space occupied by the vessel, positioned 10 mm away from the transducer array. The amplitudes of the moving and static scatterers follow Gaussian distributions with a mean of 0 and variances of 1 and 625, respectively.

Laminar blood flow in Figure 5a is modeled with a flow velocity of 0.5 m/s, and parabolic blood flow in Figure 5b is modeled with a peak flow velocity of 1.0 m/s. These images are inspired by a similar image shown on the Field II website [31]. In Field II, color Doppler imaging is performed using B-mode imaging, whereas FOCUS supports color Doppler imaging using either B-mode imaging or plane wave imaging. RF data is obtained using five plane waves steered across five evenly spaced transmit angles spanning from −6° to +6°. For delay-and-sum beamforming, the *z*-axis focal depths range from 10 mm to 80 mm in increments of 1 mm and the *x*-axis focal depths range from −20 mm to 20 mm in increments of 0.1569 mm. The f-number for the delay-and-sum beamforming is 1.5. A pulse repetition frequency of 5 kHz is used, with 50 total acquisitions—10 scans are performed at each of the five transmit angles. Flow velocities are estimated with a 1D autocorrelation phase-based estimator. Portions of the RF data with magnitudes below a 4 dB cutoff after normalization are set to zero. A Hamming moving spatial average filter is then applied with a height of 1.54 mm and a width of 1.57 mm. Figure 5 demonstrates that color Doppler imaging is effectively simulated using plane wave imaging in FOCUS. The laminar and parabolic flow profiles of the moving scatterers are evident in each image.

**Figure 6:**
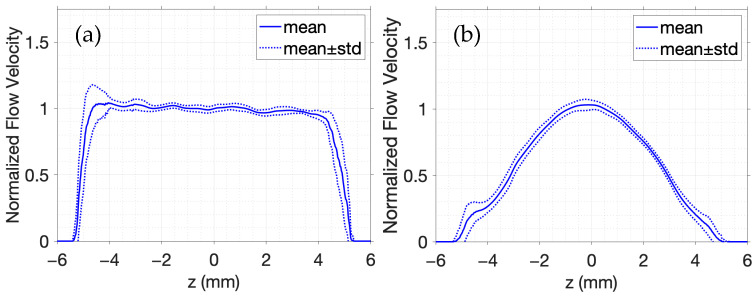
Averaged and normalized flow velocity with (**a**) laminar flow and (**b**) parabolic flow.

In Figure 6, the velocity estimates are averaged across all A-lines within the artery interior in addition to a 1 mm region immediately outside the vessel walls on either side. Each profile is normalized by the peak velocity estimate at x=0. The expected flow trends are accurately captured by Figure 6, where a flat profile in Figure 6a confirms uniform laminar flow, and a curved profile in Figure 6b reflects the parabolic velocity distribution typical of fully developed flow in a vessel. In Figure 6b, the peak velocity occurs at the vessel center and decreases toward the walls, as expected. The 1D autocorrelation phase-based estimator reliably distinguishes between the laminar and parabolic flow profiles, demonstrating the capability of simulated color Doppler imaging to describe flow patterns.

### 3.2. Model Convergence

The simulation parameters associated with Figure 2c are also evaluated to compare the convergence properties of the pulse-echo model with the fast nearfield method to the pulse-echo model with spatial impulse responses and to Field II (version 4.11, compiled for Apple Silicon Macs). For Field II simulations, the rectangular transducers of the linear array were spatially discretized by concatenating six equal-sized vertical sub-elements to facilitate comparisons with fast nearfield method calculations that incorporate six abscissas for integration. Since the pulse-echo model with the fast nearfield method is analytically equivalent to the pulse-echo model with spatial impulse responses, both are expected to ultimately converge to the same result as the temporal sampling frequency increases. Field II simulations are also analytically equivalent to pulse-echo simulations with spatial impulse responses, but Field II applies a far-field approximation. Since the simulations are conducted in the far-field, convergence to the solution obtained with the spatial impulse response is expected. The reference RF data is generated using spatial impulse responses with a temporal sampling frequency of 3.072 GHz. The RF data is computed for simulations using spatial impulse responses and Field II at temporal sampling frequencies of 96, 192, 384, and 768 MHz. Since FOCUS attains reference-level accuracy by 96 MHz, FOCUS simulations are performed at temporal sampling frequencies of 12, 24, 48, and 96 MHz. Because Field II omits the three temporal-derivative terms in Equation (1), the normal velocities $w_{1}(t)$ and $w_{2}(t)$ were differentiated prior to convolution, and the resulting RF data from Field II also underwent a differentiation to restore all three temporal-derivative contributions. Furthermore, Field II excludes the scale factor $\frac{\rho_{0}}{2c^{2}}$ from Equation (1). Therefore, the RF data from Field II was also multiplied by this scale factor to facilitate a direct comparison.

**Figure 7:**
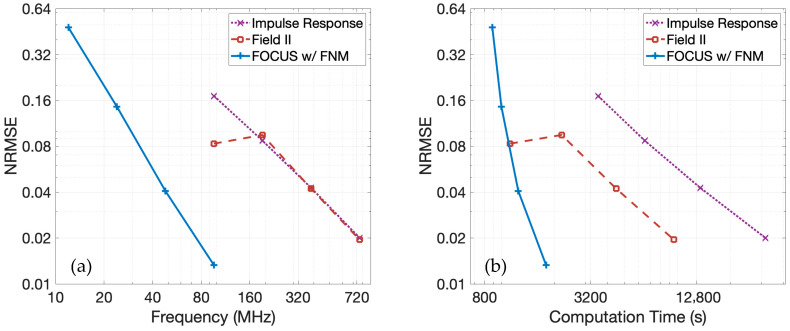
The NRMSE is shown as a function of (**a**) temporal sampling frequency and (**b**) computation time. In both figures, the NRMSE of the pulse-echo model with the fast nearfield method in FOCUS is shown by a solid blue line, the NRMSE of the pulse-echo model with spatial impulse responses is shown by a dotted purple line, and the NRMSE of Field II is shown by a dashed maroon line.

Figure 7a shows the normalized root-mean-square error (NRMSE) for each method as a function of temporal sampling frequency relative to the 3.072 GHz spatial impulse response reference. The NRMSE is evaluated over the echo-containing portions of the RF data. The pulse-echo model using the fast nearfield method, the pulse-echo model using spatial impulse responses, and the pulse-echo model with Field II all converge toward the reference solution as the temporal sampling frequency increases. However, the convergence slope is steepest for the fast nearfield method, indicating the fastest rate of convergence. Additionally, the NRMSE for the fast nearfield method is lower than that of the spatial impulse responses and Field II when these are evaluated at the same temporal sampling frequency, suggesting not only faster convergence at a given temporal sampling frequency but also superior accuracy at each temporal sampling frequency.

The NRMSE as a function of computation time is presented in Figure 7b. For all three methods, especially at higher frequencies, the computation time increases approximately linearly with increased temporal sampling frequencies. When comparing the pulse-echo model with the fast nearfield method to the pulse-echo model with spatial impulse responses, the fast nearfield method consistently achieves lower NRMSE with shorter computation times. A direct comparison between the pulse-echo model with the fast nearfield method in FOCUS and the spatial impulse response in Field II simulations reveals a distinct performance trade-off. At higher temporal sampling frequencies targeting lower NRMSE, the fast nearfield method approach is significantly faster than Field II. Both spatial impulse responses and Field II exhibit only modest error reduction with increased temporal sampling frequency, despite substantial increases in runtime, resulting in relatively shallow error–time curves. In contrast, the fast nearfield method in FOCUS generates a steep error–time curve, demonstrating that even large decreases in NRMSE require only modest additional computation time. For instance, FOCUS simulations utilizing a temporal sampling frequency of 48 MHz are completed in 20 min. To achieve comparable accuracy, Field II simulations require a much higher temporal sampling frequency of 384 MHz and about 75 min of computation time. Furthermore, when aiming for even lower errors, FOCUS simulations at 96 MHz finish in 30 min, whereas Field II requires 159 min with a temporal sampling frequency of 768 MHz. Consequently, the fast nearfield method approach also benefits from lower memory requirements, since memory scales linearly with temporal sampling frequency in these simulations.

**Figure 8:**
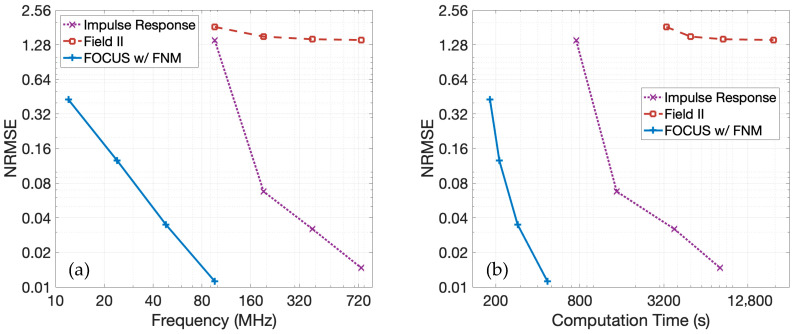
The NRMSE for nearfield simulations is shown as a function of (**a**) temporal sampling frequency and (**b**) computation time. In both figures, the NRMSE of the pulse-echo model with the fast nearfield method in FOCUS is shown by a solid blue line, the NRMSE of the pulse-echo model with spatial impulse responses is shown by a dotted purple line, and the NRMSE of Field II is shown by a dashed maroon line.

Figure 8 presents results obtained with the same transducer array and imaging parameters evaluated in Figure 7 to scatterers in the nearfield. To maintain a consistent scatterer density with the previous convergence profiling, 8333 scatterers are randomly distributed within a 50 × 10 × 5 mm region located 2 mm from the transducer array. The amplitudes of the scatterers followed a Gaussian distribution with a mean of 0 and a variance of 1. The reference RF data is again generated using spatial impulse responses at a temporal sampling frequency of 3.072 GHz.

For these calculations, the fast nearfield method requires additional abscissas for Gauss–Legendre quadrature to achieve comparable convergence. To achieve NRMSE values similar to those demonstrated in the far-field in Figure 7, 50 abscissas are defined for fast nearfield method calculations. In Field II simulations, each rectangular array element is partitioned into 50 uniform sub-elements arranged in a 2 × 25 grid.

The NRMSE for each method as a function of temporal sampling frequency relative to the nearfield reference solution is displayed in Figure 8a. The pulse-echo model using the fast nearfield method demonstrates rapid convergence as the temporal sampling frequency increases, which is consistent with the far-field results shown in Figure 7a. In contrast, spatial impulse responses and Field II converge much more slowly in the nearfield region. As a result, the fast nearfield method achieves substantially lower NRMSE values, indicating both significantly faster convergence with increased temporal sampling frequency and markedly higher accuracy in the nearfield.

Figure 8b shows the NRMSE evaluated in the nearfield region as a function of computation time. The pulse-echo model with fast nearfield method in FOCUS consistently achieves a substantially lower NRMSE with significantly shorter computation times. These results demonstrate that the pulse-echo model with fast nearfield method is well suited for nearfield simulations. In contrast, spatial impulse responses, even with many sub-elements in Field II simulations, exhibit poor convergence in the nearfield and require prohibitively high temporal sampling frequencies to achieve comparable accuracy, resulting in much longer computation times with increased memory requirements.

The parameters defined for the simulations shown in Figure 2c are again chosen to evaluate the convergence behavior of the pulse-echo model with the fast nearfield method. For this evaluation, three different approaches were employed: the fast nearfield method; the fast nearfield method with discrete-time shifting and linear interpolation; and the upsampled fast nearfield method with discrete-time shifting, linear interpolation, and downsampling prior to convolution. The reference RF data used for these comparisons is identical to the reference RF data in Figure 7, which is generated using spatial impulse responses at a temporal sampling frequency of 3.072 GHz. The RF data for each of the three approaches is then computed at temporal sampling frequencies of 12, 24, 48, and 96 MHz. Upsampled fast nearfield method calculations are computed at four times the respective temporal sampling frequencies—namely 48, 96, 192, and 384 MHz.

**Figure 9:**
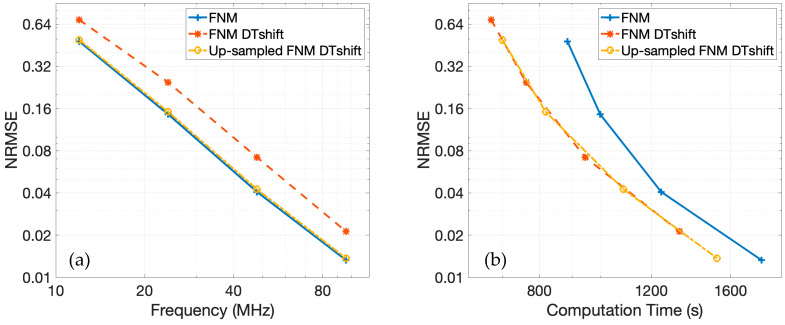
The NRMSE is shown as a function of (**a**) temporal sampling frequency and (**b**) computation time. In both figures, the NRMSE of the pulse-echo model with the fast nearfield method is shown by a solid blue line; the NRMSE of the fast nearfield method with discrete-time shifting and linear interpolation is shown by a dashed orange line; and the NRMSE of the upsampled fast nearfield method with discrete-time shifting, linear interpolation, and downsampling is shown by a dash-dotted yellow line.

Figure 9a presents the NRMSE for each of the three fast nearfield method approaches across various temporal sampling frequencies relative to the reference. The NRMSE for the baseline fast nearfield method matches the result shown in Figure 7a. Incorporating discrete-time shifting and linear interpolation introduces some additional error, as indicated by the corresponding NRMSE curve, which follows a similar convergence trend but remains consistently elevated. This error is effectively mitigated when the fast nearfield method calculations are upsampled prior to discrete-time shifting, linear interpolation, and downsampling. The NRMSE curve for this upsampled approach closely aligns with the baseline fast nearfield method approach, demonstrating that upsampling eliminates the aliasing artifacts introduced by linear interpolation.

The NRMSE as a function of computation time for the temporal sampling frequencies of 12, 24, 48, and 96 MHz across all three approaches is presented in Figure 9b. The NRMSE curves corresponding to the approaches that incorporate discrete-time shifting are shifted leftward relative to the baseline fast nearfield method approach, indicating reduced computation times. This demonstrates that discrete-time shifting effectively accelerates these simulations, particularly when simulating multiple plane wave images. Among the three approaches, the fast nearfield method with discrete-time shifting and linear interpolation yields the shortest computation times but exhibits increased error. The upsampled fast nearfield method approach offers a favorable trade-off. While slightly slower than the fastest approach, the upsampled fast nearfield method maintains NRMSE values nearly identical to those obtained by the baseline fast nearfield method. The upsampled fast nearfield method with discrete-time shifting thus achieves an appropriate balance between computational efficiency and accuracy. These results suggest that the upsampled fast nearfield method approach is best suited for efficient simulations of plane wave imaging. By combining the speed advantages of discrete-time shifting with the accuracy gains from upsampled fast nearfield method calculations, the method delivers an effective balance of accuracy and efficiency.

## 4. Discussion

### 4.1. Overview of Simulation Capabilities

The results in Figure 2, Figure 3, Figure 4, Figure 5 and Figure 6 demonstrate that FOCUS is a versatile and efficient tool for simulating several different types of ultrasound images. FOCUS now supports advanced imaging techniques such as plane wave imaging and color Doppler imaging. As illustrated in Figure 2, synthetic aperture, B-mode, and plane wave imaging are all effectively simulated with FOCUS. The pulse-echo model with the fast nearfield method handles simulations involving hundreds of thousands of scatterers without requiring a full 3D rectangular grid—a constraint that would otherwise necessitate extremely fine sampling and significantly increase the computation time. Additionally, every image consistently displays the expected background speckle pattern produced by random scatterers in soft tissue. Of particular note, the plane wave imaging simulation with 100,000 scatterers achieves high fidelity with only five steering angles and a computation time of 11 min, which is substantially faster than the 63- and 143-min simulations required for B-mode imaging and synthetic aperture imaging, respectively. Across the three images, point targets, hyperechoic regions, and anechoic regions are clearly identifiable. The results in Figure 2, Figure 3, Figure 4, Figure 5 and Figure 6 underscore the value of FOCUS simulations for accelerating the development and validation of advanced ultrasound imaging methodologies in a controlled and efficient computational framework.

FOCUS simulations are capable of modeling scenarios more complex than simple phantoms containing only point targets, hyperechoic regions, and anechoic regions. As illustrated in Figure 3 and Figure 4, FOCUS is able to replicate real-world ultrasound images. In contrast to grid-based 3D calculations, which would require days to complete on a state-of-the-art desktop computer to evaluate the same simulations, FOCUS simulations are much faster. The simulation results presented in Figure 3 were generated in just over 3.5 h, while those shown in Figure 4 required about 11.5 h. The computation time can be reduced by decreasing the total number of scatterers or by reducing the number of plane waves used in each simulation. Both examples employ a large number of frames to achieve high resolution, and the simulations exhibit an almost linear relationship between the number of scatterers/frames and the computation time. Consequently, reducing either parameter would result in a proportional decrease in computation time. Moreover, the inherent randomness incorporated into generating these simulated images allows for the creation of similar, yet distinct, ultrasound datasets [34,35]. One limitation of FOCUS imaging simulations, however, is that the pulse-echo model defined in Equation (1), which forms the basis of these simulations, is restricted to soft-tissue propagation with scattering induced by instantaneous changes in density. These assumptions preclude the simulations of high-impedance structures such as bone.

Software platforms with powerful and versatile ultrasound simulation capabilities are also applicable to education and training. By providing a controlled simulation environment, FOCUS serves as an invaluable resource for investigating ultrasound principles and exploring the effects of parameter variations. Recently, dedicated training software was developed with FOCUS to complement the book *Essentials of Ultrasound Imaging* by Szabo and Kaczkowski [36]. This book covers the core concepts and practical applications of ultrasound technology, including wave generation, tissue interaction, image processing, transducer design, beam formation, signal processing, and Doppler imaging. By strategically integrating FOCUS-based simulations, this book effectively illustrates complex ideas without requiring an extensive mathematical background. This powerful combination of theory and interactive simulation tools creates a risk-free, standardized, and highly scalable platform that provides practical experience to enhance understanding of ultrasound principles and physics.

FOCUS extends simulation capabilities beyond static imaging, enabling dynamic scenarios characterized by the complex motion of random scatterers. One example, as illustrated in Figure 5 and Figure 6, simulates color Doppler imaging of a carotid artery phantom. This example, which is now included with FOCUS, demonstrates the effective simulation of color Doppler imaging by modeling scatterers as moving particles with positions that update over time. The travel time of an acoustic wave is directly proportional to the distance between the scatterer and the transducer. Consequently, scatterer displacements between successive frames produce time shifts in the pulse-echo contributions from each scatterer to the RF data. The presented pulse-echo model for ultrasound propagation and scattering in soft tissue accurately captures scatterer motion over time and models the corresponding changes in the simulated RF data. Velocity estimates are performed through mean subtraction and a 1D autocorrelation phase-based estimator—techniques commonly applied to experimental RF data—that yield comparable outcomes on the simulated RF data. Empirical tuning identified a 4 dB cutoff threshold and a 1.54 mm × 1.57 mm Hamming moving spatial average filter as optimal for maximizing the fidelity of flow velocity estimations while suppressing both high-frequency fluctuations and contributions from static portions of the RF data. Notably, both simulations in Figure 5 and Figure 6 were each completed in just over 4 h, highlighting the computational efficiency of FOCUS in dynamic imaging simulations.

While the 1D autocorrelation phase-based estimator is effective, several inherent limitations can compromise the accuracy of the estimated velocities. One primary limitation is aliasing, which occurs when the Doppler frequency shift exceeds the Nyquist limit, resulting in wrapped or ambiguous velocity estimates. Furthermore, the angular dependency of Equation (10) means that precise velocity estimation is sensitive to the angle between the ultrasound beam and the blood flow; specifically, off-axis velocity estimates in an image can lead to systemic over- or underestimation of the blood flow velocities. Additionally, strong reflections from stationary tissue, especially prevalent near vessel walls, generate static signals that can obscure weaker flow signals, and filtering to suppress these static signals risks removing legitimate flow. Furthermore, constructive or destructive interference among backscattered echoes can produce spurious signal enhancements or cancellations, leading to overestimation, underestimation, or even complete loss of information. Additionally, other artifacts mimicking flow or obscuring flow patterns further complicate velocity estimation in color Doppler imaging. Scatterer density also plays a critical role in the accuracy of Doppler simulations. In the FOCUS simulations presented in Figure 5 and Figure 6, the number of scatterers was chosen to ensure that the density is above the conventional threshold of 10 scatterers per resolution cell [37] to ensure the formation of fully developed speckle [38]. The resulting speckle pattern provides the stability necessary for reliable flow velocity estimations using a 1D auto-correlation phase-based estimator. In contrast, insufficient scatterer representations can yield unrealistic speckle patterns and degraded estimation accuracy. To address these many limitations, more advanced estimation techniques are actively being researched for quantitative blood-flow analysis. In this context, FOCUS simulations provide an invaluable resource to assist in the development and validation of these novel techniques.

### 4.2. Comparisons to Other Methods and Simulation Tools

Compared to the pulse-echo model with spatial impulse responses and Field II simulations, the pulse-echo model with the fast nearfield method achieves faster convergence and reduced computation times, especially for low-NRMSE simulations. Improved accuracy is evident, especially in the nearfield region. Figure 7 and Figure 8 show that increasing the temporal sampling frequency rapidly drives the convergence of the pulse-echo model with the fast nearfield method, whereas the convergence of the pulse-echo model with spatial impulse responses and Field II lag considerably. As a result, high-quality images are generated much more quickly using the pulse-echo model with the fast nearfield method.

The pulse-echo model with the upsampled fast nearfield method, discrete-time shifting, linear interpolation, and downsampling demonstrates the best performance overall in most cases. Pulse-echo signals, which are typically smooth and well-behaved when sufficiently sampled, allow linear interpolation to maintain high accuracy with regard to temporal alignment while introducing minimal errors in signal amplitude. Upsampling further reduces errors caused by aliasing during linear interpolation. This approach achieves accuracy comparable to the fast nearfield method with no discrete time shifting or upsampling while significantly reducing computation times.

When compared to other widely used ultrasound simulation tools, FOCUS presents several distinct advantages. Spatial impulse response-based methods, for instance, often require very high temporal sampling frequencies to ensure convergence, resulting in increased computational costs. Many sub-elements are also required to approximate circular or irregular shapes. When implemented in the frequency domain, the spatial impulse response encounters difficulties with Gibbs oscillations, which restricts the range of excitation functions that can be simulated. Additionally, the reliance on high temporal sampling frequencies in impulse response-based simulations also leads to increased memory requirements.

In Figure 7 and Figure 8, FOCUS is also compared with Field II. Both FOCUS and Field II employ a time-domain approach to the same underlying pulse-echo model shown in Equation (1) and thus share a common analytical foundation. This commonality allows for a fair assessment of algorithmic efficiency and accuracy. In contrast, simulators like k-Wave and FullWave, which solve the wave equation on structured meshes via finite-difference or pseudospectral methods, deploy fundamentally different algorithms and require substantially longer runtimes, making a fair and practical comparison challenging.

The results from Figure 7 and Figure 8 demonstrate the advantages of FOCUS compared to Field II. In Field II simulations, common practice involves avoiding temporal sampling frequencies below about 100 MHz due to the high error rates encountered at lower frequencies. The overall trend is that as the temporal sampling frequency increases, the performance of FOCUS surpasses the performance of Field II across all metrics, including error, computation time, and memory usage. Specifically, FOCUS simulations with a temporal sampling frequency of 48 MHz demonstrate an error comparable to Field II simulations with a temporal sampling frequency of 384 MHz. This means Field II requires an eight-fold higher temporal sampling frequency to achieve accuracy comparable to FOCUS at 48 MHz. In terms of computation time, FOCUS completes the 48 MHz simulation in 20 min, compared to 75 min for the Field II simulation at 384 MHz, representing a 3.75× speedup for FOCUS. The elevated temporal sampling frequency for Field II also incurs an eight-fold increase in memory usage. Similarly, for higher precision simulations, FOCUS at 96 MHz achieves an error comparable to Field II at 768 MHz. Again, Field II necessitates an eight-fold greater temporal sampling frequency. In that case, the FOCUS simulation takes 30 min compared to 159 min for the Field II simulation, resulting in a 5.3× speedup for FOCUS. An eight-fold greater memory demand is again incurred by the Field II simulation. Interestingly, the NRMSE values produced by Field II are comparable for the temporal sampling frequencies of 96 and 196 MHz, which suggests that the Field II solution is experiencing a change in the rate of convergence within this frequency range. With an NRMSE of approximately 0.1, a visual inspection of the generated simulated images reveals noticeable method-generated artifacts. For most applications, these artifacts are undesirable, which motivates temporal sampling frequencies of 348 MHz or more for Field II simulations. Collectively, the fast nearfield method approach implemented in FOCUS offers a significant computational advantage. This is especially evident in nearfield simulations, where FOCUS simulations greatly outperform Field II simulations across all of the discussed metrics.

Beyond these direct comparisons, FOCUS offers additional advantages. FOCUS employs fast nearfield method solutions for rectangular, circular, spherical, flat ring, and spherical ring transducers, eliminating the need to approximate common transducer shapes with sub-elemental rectangular or triangular elements. Moreover, the fast nearfield method converges at lower temporal sampling frequencies, reducing the computational burden compared to spatial impulse response-based methods. This lower temporal sampling frequency requirement not only decreases computation time but also directly translates to lower memory requirements for FOCUS simulations. Beyond efficiency, FOCUS supports any smooth excitation function, models several different transducer geometries, and defines physically meaningful units for the RF data and pressure field computations. These advantages are achieved in FOCUS while avoiding Gibbs oscillations.

FOCUS models each pulse-echo signal as an interaction between one transmitting transducer, one scatterer, and one receiving transducer. The complete assembly of the RF data is obtained by summing the pulse-echo signals from all such interactions according to the requirements of the desired imaging method. Consequently, FOCUS imaging simulations readily extend to 2D arrays and other custom transducer configurations. By building upon the provided example scripts, users of FOCUS can implement sophisticated array designs and alternative imaging methods with minimal customization.

Finally, while finite difference and the pseudo-spectral method have certain benefits, the high computational cost contrasts sharply with the efficiency of FOCUS, which performs simulations in a fraction of the time with a fraction of the computational resources. FOCUS offers a robust framework that combines high accuracy, flexibility, and computational efficiency, making FOCUS well-suited for a wide range of ultrasound applications.

### 4.3. Future Work and Other Considerations

All of the FOCUS imaging simulations shown in Figure 2, Figure 3, Figure 4, Figure 5, Figure 6, Figure 7, Figure 8 and Figure 9 were conducted using a single CPU core. With computation times ranging from minutes to hours, parallel acceleration was not needed for any of the simulation scenarios shown. Nevertheless, a significant increase in the number of frames, scatterers, or transducers could render serial computation times impractical, thereby motivating parallelization. GPUs are particularly well-suited for FOCUS simulations because the underlying computations are embarrassingly parallel at multiple levels. This allows for numerous independent calculations to be performed simultaneously, which is ideal for massive parallel processing. However, prior work on transient fast nearfield method computations has indicated that memory bandwidth and data-transfer delays can significantly hinder parallel efficiency when utilizing consumer-class GPUs [39]. Significant advancements in data center-class GPUs, such as the NVIDIA A100, H100, and H200, offer substantial promise in overcoming these challenges. These advanced GPUs are expected to achieve great improvements in parallel efficiency due to significantly higher memory bandwidths and data-transfer rates. With these hardware improvements, GPU-accelerated FOCUS simulations are expected to achieve excellent parallel performance, enabling the efficient simulation of large-scale configurations that were previously computationally prohibitive. To realize these improvements, formulating the programmatic framework for GPU-based FOCUS simulations is necessary, followed by empirically validating the performance gains.

The results show that FOCUS imaging simulations are effective for modelling static phantoms, realistic tissue environments, and dynamic scatterer motion. However, FOCUS simulations are also applicable to other scenarios. For instance, the pulse-echo model at the core of FOCUS imaging simulations can generate the RF data for methods such as shear-wave elastography [40,41] and super-resolution ultrasound imaging [42,43]. These require additional modeling and analysis steps beyond the generation of RF data but are nevertheless topics of great interest. For shear-wave elastography, for example, simulation of tissue mechanical response and shear-wave propagation is required prior to the generation of RF data, and the processing of the RF data is also more involved. Similarly, super-resolution imaging depends on velocity estimation via more elaborate post-processing techniques. As additional capabilities are developed, the existing framework in FOCUS is expected to serve as the foundation for future investigations into these and other emerging ultrasound imaging techniques.

## 5. Conclusions

FOCUS provides a versatile and efficient simulation framework for ultrasound imaging. FOCUS is capable of simulating conventional techniques such as synthetic aperture imaging, B-mode imaging, plane wave imaging, and color Doppler imaging. On a standard personal or desktop computer, plane wave imaging simulations involving hundreds of thousands of scatterers are performed in minutes. More complex plane wave imaging simulations, involving millions of scatterers and many steering angles, are completed in a few hours. The computation times of synthetic aperture and B-mode imaging simulations also typically range from minutes to hours, depending on the simulation parameters. Plane wave imaging simulations yield high-fidelity beamformed images with only a few steering angles, while color Doppler simulations reproduce scatterer motion to enable flow velocity estimation from simulated RF data. Realistic speckle patterns and characteristic image features, such as hyperechoic and anechoic regions, as well as more complex anatomical structures, are faithfully reproduced in FOCUS imaging simulations. In both the nearfield and far-field regions, the pulse-echo model with the fast nearfield method converges more rapidly and achieves greater accuracy than the pulse-echo model with spatial impulse responses and Field II. Support for diverse probe geometries and arbitrary excitation signals further extends the capabilities of FOCUS simulations. Such capabilities position FOCUS as a robust and efficient platform for advancing ultrasound imaging technology, providing researchers with a powerful tool for testing, validating, and refining both established and novel imaging techniques.

## Data Availability

The original contributions presented in this study are included in the article. Further inquiries can be directed to the corresponding author.
